# Opioid combination for cancer pain

**DOI:** 10.1038/sj.bjc.6601806

**Published:** 2004-04-13

**Authors:** S Mercadante

**Affiliations:** 1 Pain Relief & Palliative Care Unit, La Maddalena Cancer Center, Via San Lorenzo 312, Palermo 90146, Italy

**Sir**,

I read with interest the paper by Lauretti *et al* (2003) regarding the possible advantage of combining opioids in cancer pain management. They report in a crossover study that the morphine consumption given as rescue doses of immediate release morphine was lower in the oxycodone phase rather than during morphine phase. Moreover, nausea and vomiting were less frequent in oxycodone phase. While the idea of combining opioids is fascinating, data reported can merely be the result of the conversion ratio used between oxycodone and morphine, which remains unclear, and/or of the individual response when switching from one drug to another during crossover. This may occur in patients treated with a certain drug and receiving another drug as needed, for which they are less tolerant, or in other terms more responsive. Of course, nausea and vomiting will be more frequently observed in patients who will require more extra doses of morphine.

On the other hand, combination of opioids can be really effective for other reasons, other than the obvious disparity in effecting different receptor subgroups and specificity, as reported to explain the benefits of opioid switching, Anecdotally, multiple opioids are often simultaneously administered for different reasons, although it should be considered a nonsense approach. However, recent experimental data have offered a rationale for using different opioids in an attempt to improve the analgesia or to limit the development of tolerance in difficult conditions such as the need for rapid escalation. Authors report experimental studies in animals where subantinociceptive doses of oxycodone were given with morphine, resulting in a synergic analgesic effect, but in their study they are administering presumably equianalgesic doses of the two opioids (Ross *et al*, 2000). This observation seems to be attributed to a significant variability among opioid drugs in inducing rapid endocytosis of opioid receptors. This is an independent functional property that distinguishes clinically opioid drugs. Recently, the regulation of opioid receptors by endocytosis has been hypothesised to have protective functions in reducing the development of tolerance. Agonist activity and receptor endocytosis have opposing effects on receptor-mediated signalling, and the final result is a function of both processes (named RAVE). Morphine, in comparison with other opioids, has a high activity-endocytosis ratio, and has an enhanced propensity to prolonging signals with prolonged drug exposure. Molecular events, such as desensitisation and endocytosis, would reduce this response. It has been experimentally demonstrated that endocytosis-promoting agonists may facilitate morphine-induced receptor endocytosis, reducing the compensatory adaptive cellular changes that lead to upregulation of the cAMP pathway (He *et al*, 2002). Thus, a combination of opioids with different characteristics may reciprocally alter their RAVEs, so reducing the potential for the development of tolerance. In the clinical setting, the administration of small doses of a second opioid in patients with an unfavourable response during escalation with the prior opioid has been found effective in a preliminary experience, not yet published by our group. According to these observations, opioid ‘semiswitching’ could be a new therapeutic option in patients requiring escalating doses of opioids. A better knowledge of molecular mechanisms that modulate the opioid response may offer alternative treatments, which should be tested in the clinical setting. These data, supported by recent molecular investigation, should be considered preliminary and should be confirmed in a larger number of patients in controlled studies.
